# Evaluation of the Cytotoxic and Antimigratory Activity Induced by [Pt(1-hexyl-1*H*-imidazole)(η^1^-C_2_H_4_OEt)(phen)]Cl in Pancreatic Ductal Adenocarcinoma Cells

**DOI:** 10.3390/ijms27146315

**Published:** 2026-07-16

**Authors:** Gianluca Rovito, Erika Stefàno, Asjad Ali, Danilo Migoni, Federica De Castro, Antonella Muscella, Francesco Paolo Fanizzi, Michele Benedetti, Santo Marsigliante

**Affiliations:** Department of Biological and Environmental Sciences and Technologies, University of Salento, 73100 Lecce, Italyerika.stefano@unisalento.it (E.S.); asjad.ali@unisalento.it (A.A.); federica.decastro@unisalento.it (F.D.C.); fp.fanizzi@unisalento.it (F.P.F.); michele.benedetti@unisalento.it (M.B.); santo.marsigliante@unisalento.it (S.M.)

**Keywords:** pancreatic ductal adenocarcinoma, platinum (II) complex, cisplatin, apoptosis, antimigratory activity, chemoresistance

## Abstract

Pancreatic ductal adenocarcinoma (PDAC) is a highly aggressive malignancy characterized by poor prognosis and marked resistance to chemotherapy. In this study, we investigated the cytotoxic and antimigratory effects of a cationic monofunctional organometallic platinum (II) complex containing 1,10-phenanthroline (phen), [Pt(1-hexyl-1*H*-imidazole)(η^1^-C_2_H_4_OEt)(phen)]Cl, in comparison with cisplatin in the cisplatin-resistant YAPC pancreatic cancer cell line. The complex exhibited a rapid and potent cytotoxic effect, significantly reducing cell viability within a few hours of treatment and showing greater short-term activity than cisplatin. This enhanced efficacy was associated with a markedly higher and faster intracellular accumulation, suggesting improved cellular uptake. Mechanistically, the compound induced apoptosis more effectively than cisplatin, as demonstrated by flow cytometry, and caused an early and pronounced loss of mitochondrial membrane potential (ΔΨ*_M_*), indicating mitochondrial involvement in cell death. In addition, the complex significantly impaired cell motility in both transwell and 3D spheroid-based assays, confirming a strong antimigratory and anti-dissemination potential. Overall, these findings indicate that [Pt(1-hexyl-1*H*-imidazole)(η^1^-C_2_H_4_OEt)(phen)]Cl represents a promising candidate for targeting cisplatin-resistant PDAC cells, owing to its rapid cellular uptake, mitochondrial-mediated apoptotic signaling, and combined cytotoxic and antimigratory properties.

## 1. Introduction

Pancreatic ductal adenocarcinoma (PDAC) is one of the most aggressive and therapeutically challenging human malignancies, accounting for more than 90% of pancreatic cancer cases and representing a major cause of cancer-related mortality worldwide [1,2]. Due to its asymptomatic onset, rapid metastatic dissemination, extensive stromal desmoplasia, and limited response to conventional therapies, PDAC remains associated with an extremely poor prognosis [3,4]. Epidemiological studies indicate that pancreatic cancer is among the leading causes of cancer-related death and is expected to represent an increasing global health burden in the coming decades [5,6,7]. Despite advances in diagnostic strategies and a growing understanding of the molecular mechanisms underlying disease progression, most patients are diagnosed at advanced stages when surgical resection is no longer feasible, resulting in a 5-year survival rate below 10% [2,8].

Chemotherapy therefore remains a cornerstone of PDAC management. Gemcitabine, approved by the U.S. Food and Drug Administration in 1996, continues to represent a standard therapeutic option, either alone or in combination regimens [9]. Platinum-based agents, particularly cisplatin, have been extensively used because of their ability to induce DNA damage, cell-cycle arrest, and apoptosis in tumor cells [10,11,12]. Cisplatin enters cells through both passive diffusion and transporter-mediated mechanisms and exerts its cytotoxic activity primarily through the formation of DNA adducts that interfere with replication and transcription processes [13,14]. Several studies have shown that cisplatin-containing regimens may improve therapeutic outcomes in PDAC, especially when administered in combination with gemcitabine [15]. However, its clinical efficacy is frequently limited by the emergence of intrinsic and acquired resistance mechanisms, including enhanced DNA repair, altered drug transport, activation of survival signaling pathways, and impaired apoptotic responses [16,17,18].

These limitations have stimulated the development of novel platinum-based compounds with improved pharmacological properties and alternative mechanisms of action [19,20,21]. Among these, organometallic platinum complexes have attracted considerable attention because their structural versatility allows modulation of cellular uptake, intracellular targeting, and antitumor selectivity [14,19]. Several phenanthroline-containing platinum (II) complexes have also demonstrated promising antitumor activity while exhibiting reduced toxicity toward non-malignant cells, supporting the therapeutic potential of this class of compounds [22]. Moreover, platinum (II) complexes incorporating specific targeting strategies have shown the ability to inhibit tumor cell proliferation and migration through multiple intracellular mechanisms [23].

Previous studies from our group demonstrated that platinum (II)-based organometallic complexes containing 1,10-phenanthroline exert potent cytotoxic effects against chemoresistant pancreatic cancer cell lines, supporting the therapeutic potential of this class of compounds [24].

Given that resistance to conventional platinum-based therapies represents a major obstacle to effective PDAC treatment [1,4], compounds previously characterized in other tumor models that exhibit favorable selectivity and enhanced cytotoxic activity may represent promising candidates for evaluation in this treatment-refractory malignancy [19,20,21]. Although initially characterized in renal carcinoma models, the physicochemical properties and selectivity profile of Pt(HexIm)(phen) suggest potential activity across highly chemoresistant tumor types, such as PDAC [25].

Pt(HexIm)(phen) is a cationic monofunctional organometallic platinum (II) complex containing two biologically relevant ligands, 1,10-phenanthroline (phen) and 1-hexylimidazole. Imidazole is a five-membered nitrogen-containing heterocycle widely present in biologically active molecules and pharmaceutical compounds. Owing to its versatile electronic properties and its ability to establish multiple non-covalent interactions with biomolecular targets, the imidazole scaffold has been associated with diverse biological activities, including antimicrobial, antifungal, and anticancer effects [26,27]. A previous study conducted by our group demonstrated that Pt(HexIm)(phen) exhibited greater in vitro cytotoxic activity than cisplatin in the human renal carcinoma cell line Caki-1 while showing lower toxicity toward non-tumoral HK-2 renal epithelial cells, indicating a more favorable selectivity profile [25]. The enhanced biological activity of this compound may be related, at least in part, to its rapid intracellular accumulation, which has been associated with the presence of the phenanthroline ligand and the organometallic structure of the complex.

Based on these findings, the present study evaluated the cytotoxic activity of Pt(HexIm)(phen) across three PDAC cell lines (BxPC-3, Mia PaCa-2, and YAPC), selected to represent distinct molecular backgrounds and different intrinsic sensitivities to cisplatin. Previous studies have shown that PDAC cell lines display heterogeneous responses to cisplatin, with BxPC-3 and Mia PaCa-2 generally exhibiting higher sensitivity, whereas YAPC cells are more resistant, in part due to mesenchymal features and genetic alterations, including KRAS mutations [18,28,29,30]. Moreover, differential cisplatin responses between BxPC-3 and YAPC cells have been associated with ERK1/2 pathway activation, suggesting a role for this signaling axis in intrinsic resistance mechanisms [31].

Following this comparative cytotoxic screening, which identified YAPC cells as the most chemoresistant phenotype, further functional analyses were focused on this cell line to evaluate the potential of Pt(HexIm)(phen) to overcome cisplatin insensitivity. Specifically, we investigated the cytotoxic, antimigratory, and pro-apoptotic effects of Pt(HexIm)(phen) and compared its activity with that of cisplatin.

## 2. Results

### 2.1. Comparative Cytotoxicity of Cisplatin and [Pt(1-hexyl-1H-imidazole)(η^1^-C_2_H_4_OEt)(phen)]Cl in Pancreatic Cancer Cell Lines

**Figure 1:**
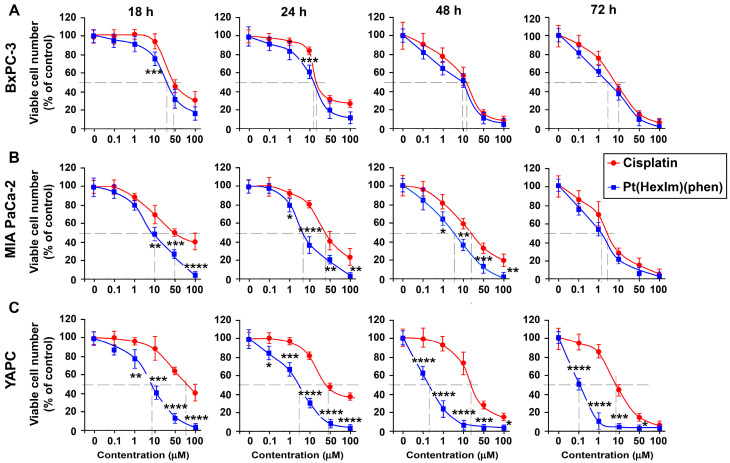
Cytotoxic effects of cisplatin and Pt(HexIm)(phen) on pancreatic tumor lines. BxPC-3 (**A**), Mia Paca-2 (**B**), and YAPC (**C**) cells were treated with different concentrations of cisplatin or Pt(HexIm)(phen) (0.1–100 µM), and cell viability was measured with sulforhodamine B (SRB) colorimetric assay, after 18, 24, 48, and 72 h. Data are the means ± standard deviation (SD) of three independent experiments with eight replicates in each and are presented as a percentage of control at the corresponding time point, with the control set as 100%. Asterisks indicate significantly different values (* *p* < 0.05; ** *p* < 0.01; *** *p* < 0.001; **** *p* < 0.0001) compared to cisplatin at the same concentration. The dashed lines indicate the IC_50_ values.

To validate the antitumor activity of Pt(HexIm)(phen) across pancreatic ductal adenocarcinoma (PDAC) models with different intrinsic sensitivities to cisplatin, we compared its cytotoxic effects in three PDAC cell lines (BxPC-3, Mia PaCa-2, and YAPC). These cell lines have previously been characterized by distinct genotypic and phenotypic features and different responses to cisplatin, with BxPC-3 and Mia PaCa-2 being relatively sensitive and YAPC displaying a more resistant phenotype [31]. Cell viability was assessed after exposure to increasing concentrations (0.1–100 μM) of cisplatin or Pt(HexIm)(phen) for 18, 24, 48, and 72 h using the sulforhodamine B (SRB) assay (Figure 1).

Treatment with Pt(HexIm)(phen) induced a concentration- and time-dependent reduction in cell viability in all three pancreatic cancer cell lines, although both the magnitude and kinetics of the response varied according to the cellular model. Pt(HexIm)(phen) consistently exhibited greater cytotoxic activity than cisplatin in the YAPC cells, with significant differences observed even at low micromolar concentrations (*p* < 0.0001). Similarly, Mia PaCa-2 cells showed a significantly greater response to Pt(HexIm)(phen) than to cisplatin, especially at the earlier time points (18–48 h). In contrast, BxPC-3 cells were less responsive to both treatments, and the differences between the two compounds progressively diminished over time.

Accordingly, further analyses were focused on the YAPC cell line, a well-established model of cisplatin-resistant PDAC, to characterize in greater detail the biological effects of Pt(HexIm)(phen).

**Figure 2:**
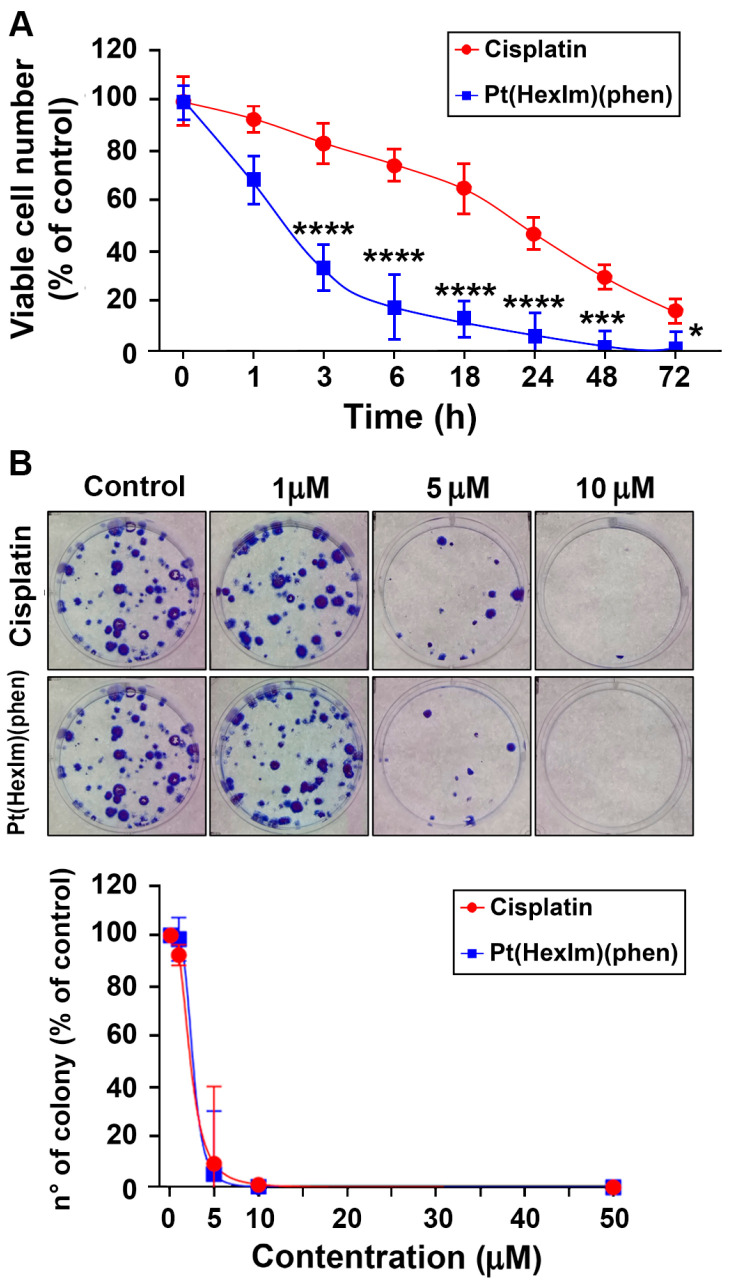
Time-dependent cytotoxicity and long-term clonogenic effects of Pt(HexIm)(phen) and cisplatin in YAPC cells. (**A**) YAPC cells were treated with 50 µM cisplatin or Pt(HexIm)(phen), and cell viability was assessed by the sulforhodamine B (SRB) assay after the indicated incubation times. Data are expressed as the mean ± standard deviation (SD) of three independent experiments, each performed with eight replicates, and are presented as the percentage of the corresponding untreated control (set at 100%). Asterisks indicate significant differences between Pt(HexIm)(phen) and cisplatin at the same concentration and time point (* *p* < 0.05; *** *p* < 0.001; **** *p* < 0.0001). (**B**) Long-term cytotoxicity was evaluated by a clonogenic survival assay. YAPC cells were exposed for 1 h to 1, 5, 10 and 50 µM cisplatin or Pt(HexIm)(phen). After 15 days, colonies containing more than 50 cells were counted. Colony numbers are expressed as the mean ± SD of three independent experiments conducted using duplicate samples.

To compare the temporal profile of cytotoxicity under identical exposure conditions, YAPC cells were treated with 50 µM Pt(HexIm)(phen) or 50 µM cisplatin for 1, 3, 6, 18, 24, and 48 h. Pt(HexIm)(phen) induced a significantly earlier and greater reduction in cell viability than cisplatin throughout the time course (Figure 2A), indicating a more rapid onset of cytotoxic activity.

Long-term cytotoxic activity was assessed by a clonogenic assay. Both cisplatin and Pt(HexIm)(phen) significantly reduced colony formation at 5 and 10 μM, while no significant effects were observed at 1 μM (Figure 2B). Although Pt(HexIm)(phen) exhibited greater short-term cytotoxicity, both compounds produced a comparable inhibition of clonogenic survival at the concentrations tested, suggesting that their long-term antiproliferative effects converge over time.

### 2.2. Intracellular Uptake of Pt Assessed by ICP-AES

**Figure 3:**
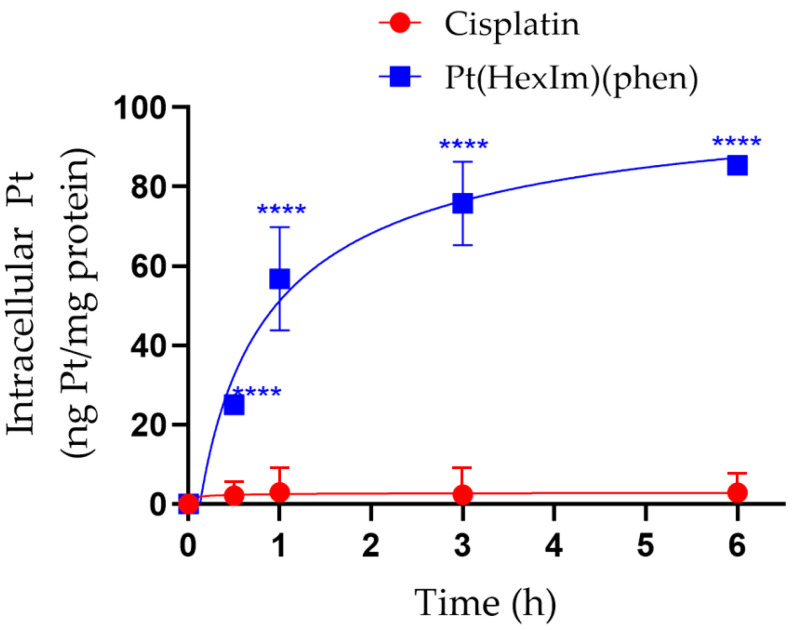
Intracellular uptake of Pt(HexIm)(phen) and cisplatin in YAPC cells. Cells were exposed to 50 μM of cisplatin and Pt(HexIm)(phen) for 0.5, 1, 3, and 6 h. Total intracellular accumulation was measured by inductively coupled plasma atomic emission spectroscopy (ICP-AES). Each data point represents the mean ± SD of three independent experiments and is expressed as ng Pt(II)/mg protein. Asterisks indicate significantly different values (**** *p* < 0.0001) compared to cisplatin at the same concentration.

Total intracellular Pt levels in YAPC cells were measured by ICP-AES following incubation with 50 μM cisplatin and Pt(HexIm)(phen) for 0.5, 1, 3, and 6 h. The complex exhibited rapid cellular uptake, with significantly higher intracellular levels than cisplatin within 30 min (*p* < 0.05), as shown in Figure 3. After 6 h, accumulation was approximately 40-fold greater than cisplatin. These data indicate that enhanced cytotoxicity is closely associated with increased intracellular accumulation.

### 2.3. Pt(HexIm)(phen) Induces Apoptosis on YAPC Cells

**Figure 4:**
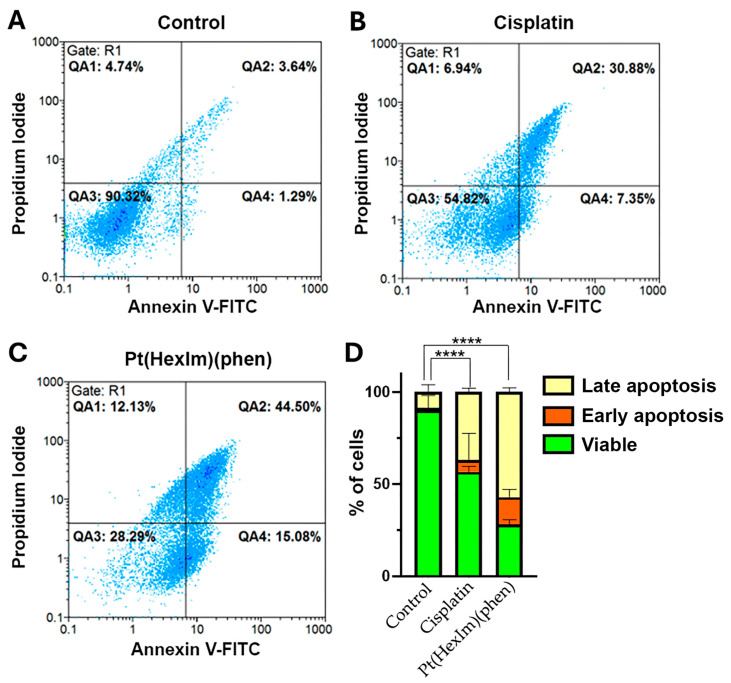
Apoptosis of YAPC cells was quantified by flow cytometry after staining with annexin V-FITC/PI. Cells were treated without (**A**) or with 50 μM (**B**) cisplatin or 10 μM (**C**) Pt(HexIm)(phen) for 24 h. QA1, PI^+^ (necrotic cells); QA2, annexin V-FITC + PI^+^ (late apoptotic and necrotic cells); QA4, annexin V-FITC + PI− (early apoptotic cells); QA3, annexin V-FITC − PI− (live cells). (**D**) The percentage of viable and apoptotic cells was quantified using FloMax software version 2.3 and displayed as a bar graph. Each data point represents the mean ± SD of three independent experiments. Asterisks (**** *p* < 0.0001) indicate viable cell values that are significantly different from control after exposure to cisplatin and Pt(HexIm)(phen).

The effects of Pt(II) complexes on apoptosis in YAPC cells were evaluated by flow cytometry using Annexin V-FITC/PI staining after 24 h of treatment. Exposure to both cisplatin and Pt(HexIm)(phen) significantly increased apoptosis compared to untreated controls (*p* < 0.0001). Notably, the imidazole-containing complex induced a greater extent of cell death than cisplatin (*p* < 0.05). Apoptotic/necrotic cells increased from 8.6% in controls to 37% and 57.1% following cisplatin and complex treatment, respectively (Figure 4).

**Figure 5:**
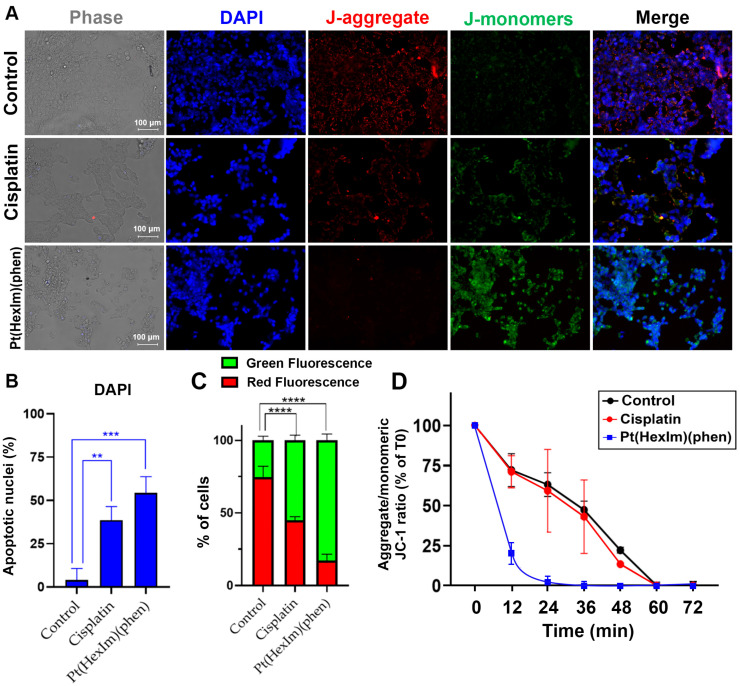
Analysis of mitochondrial membrane potential using the cationic dye JC-1 in YAPC cells. (**A**) Double staining of nuclei (DAPI) and mitochondria (JC-1) was assessed in YAPC cells by fluorescence microscopy (ladder: 100 μm). Cells were incubated or not with 50 μM cisplatin and 10 μM Pt(HexIm)(phen) for 24 h and stained with DAPI and JC-1 dyes. Quantification of green/red fluorescence (**A**,**C**) for measurement of mitochondrial membrane potential (ΔΨ*_M_*) and blue fluorescence (**A**,**B**) for apoptotic nuclei was performed using ImageJ software version 1.54t. Data are represented as the mean ± SD from three independent experiments. Asterisks (** *p* < 0.01; *** *p* < 0.001; **** *p* < 0.0001) indicate significantly higher values compared to untreated cells. (**D**) Fluorescence measurements of J aggregate and J monomer were also obtained by fluorimetry. Data are expressed as the change in the fluorescence ratio at 590/520 nm induced by treatment compared to the initial (control) ratio at 590/520 nm. Results are presented as the mean ± SD of three independent experiments.

Mitochondrial membrane potential (ΔΨ*_M_*) is a key indicator of mitochondrial function and cellular health. In viable cells, high ΔΨ*_M_* promotes the formation of JC-1 aggregates, which emit red fluorescence, whereas in apoptotic cells, reduced ΔΨ*_M_* results in JC-1 monomers emitting green fluorescence. The red/green fluorescence ratio therefore reflects changes in ΔΨ*_M_* and the onset of apoptosis. ΔΨ*_M_* was assessed by fluorescence microscopy (JC-1/DAPI staining, Figure 5A–C) and by fluorometric analysis in the early phases of treatment (Figure 5D). Double staining revealed a significant increase in apoptotic (green fluorescent) cells following treatment with both compounds, with a more pronounced effect observed for Pt(HexIm)(phen) (*p* < 0.0001). Fluorometric analysis confirmed an early decrease in ΔΨ*_M_*, detectable as soon as 12 min after treatment with the complex, whereas cisplatin induced a slower and less pronounced effect.

**Figure 6:**
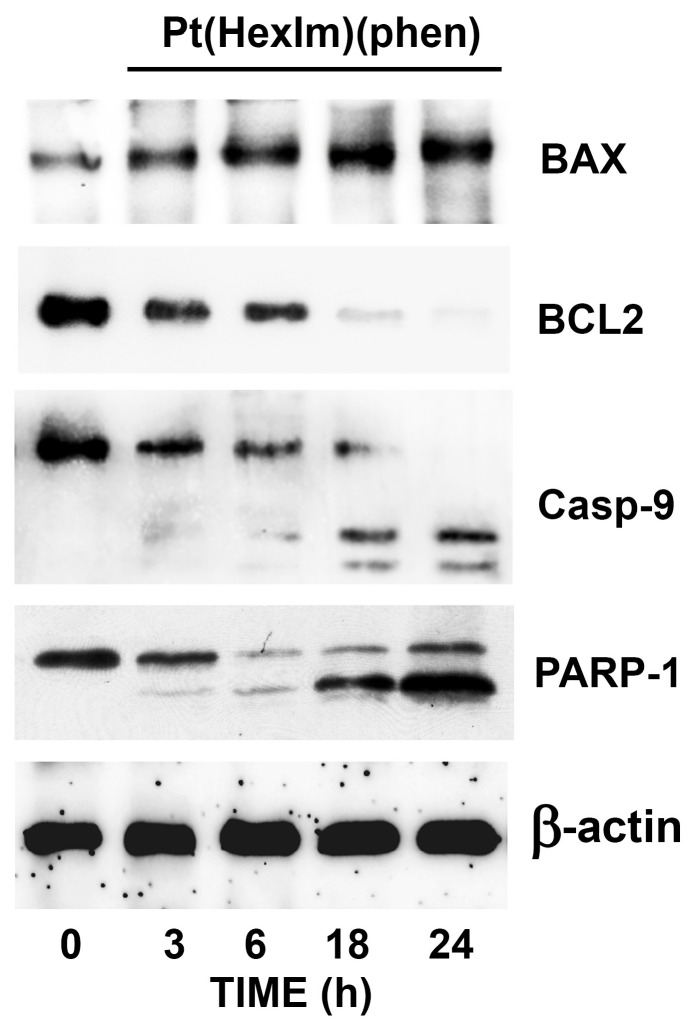
Effects of Pt(HexIm)(phen) on the expression of apoptosis-related proteins in YAPC cells. Cells were treated with the platinum complex and harvested at the indicated time points (0, 3, 6, 18, and 24 h). Total cell lysates were analyzed by Western blotting for BAX, BCL2, Caspase-9 (Casp-9), and PARP-1. β-actin was used as a loading control. Blots are representative of three independent experiments.

To further elucidate the molecular mechanisms driving the intrinsic apoptotic pathway, the expression of key apoptosis-regulatory proteins was evaluated by Western blot analysis over a 24 h time course (Figure 6). Treatment with Pt(HexIm)(phen) induced a time-dependent upregulation of the pro-apoptotic protein BAX, accompanied by a progressive down-regulation of the anti-apoptotic protein BCL2, resulting in an increased BAX/BCL2 ratio. Consistently, the activation of the mitochondrial pathway was confirmed by the time-dependent cleavage and activation of the initiator Caspase-9, visible starting from 6 h. This enzymatic cascade culminated in the efficient cleavage of the downstream executioner caspase substrate, PARP-1, within the same timeframe (Figure 6). Together, these molecular changes confirm that the rapid mitochondrial depolarization induced by the complex triggers a canonical intrinsic apoptotic cascade.

### 2.4. Pt(HexIm)(phen) Reduces YAPC Cells Migration

**Figure 7:**
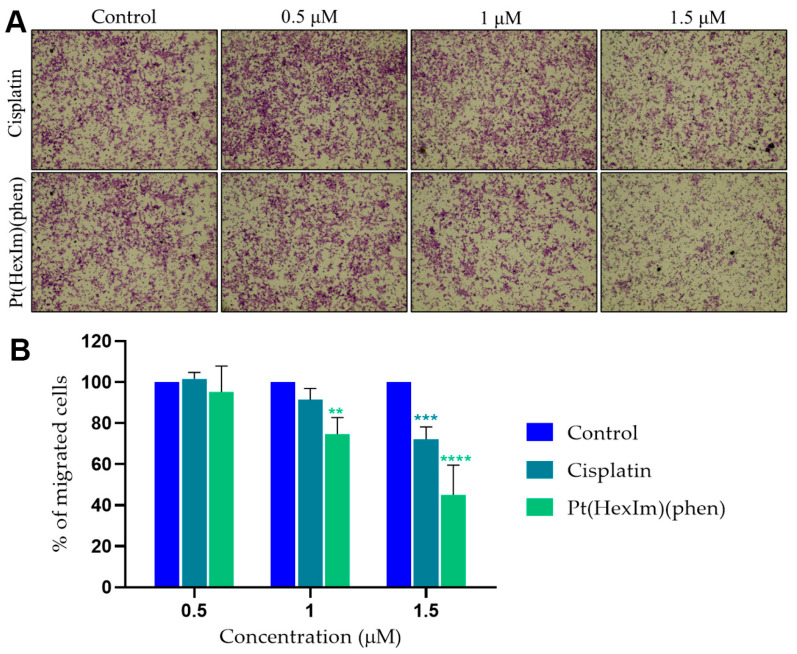
Antimigratory capacity of Pt(II) complexes on YAPC cells. The migratory capacity of YAPC cells was assessed using the trans well chemotaxis assay after exposure to sublethal doses of cisplatin and Pt(HexIm)(phen). (**A**) Cells were treated with different concentrations (0.5, 1, and 1.5 μM) of Pt(II) compounds for 24 h and monitored by microscopy. (**B**) The percentage of cells that migrated after treatment was quantified at 24 h and normalized to the control. Images were collected using a microscope (Evos XL, Core; Invitrogen) with a 10× objective. Data are expressed as mean ± SD of three independent experiments, each performed in duplicate. (** *p* < 0.01, *** *p* < 0.001, **** *p* < 0.0001 by one-way ANOVA; *n* = 3).

Cell migration was evaluated using transwell and 3D spheroid assays following treatment with sublethal concentrations (0.5–1.5 μM). Pt(HexIm)(phen) markedly inhibited migration in transwell assays, reducing cell motility by ~50% at the highest concentration (*p* < 0.0001), with a weaker effect observed for cisplatin (*p* < 0.001) (Figure 7).

**Figure 8:**
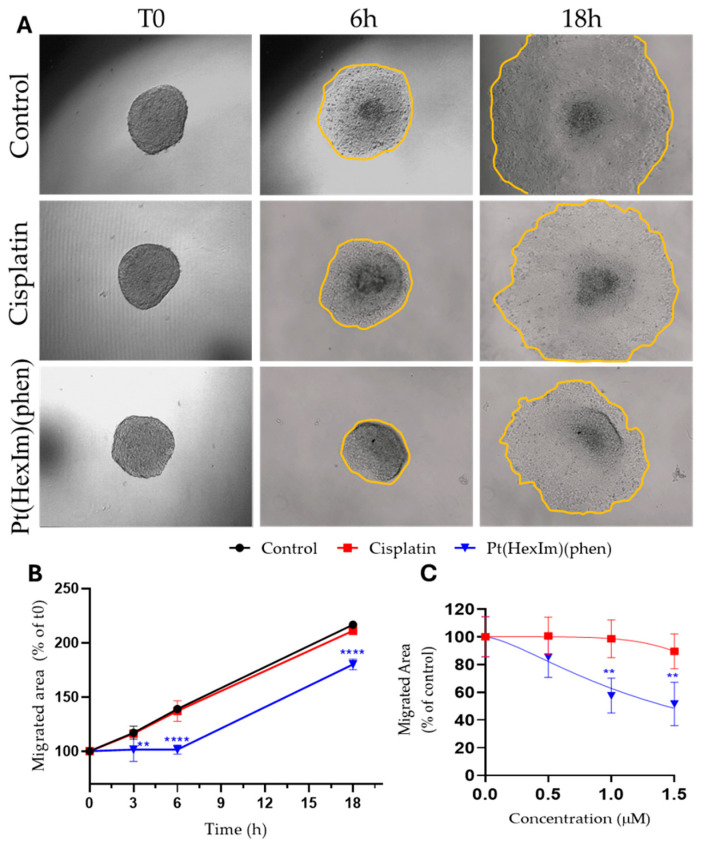
Effects of cisplatin and Pt(HexIm)(phen) on antimigratory capacity. (**A**) Tumor spheroids were transferred to a 96-well flat-bottom migration plate and treated with or without 0.5, 1, and 1.5 μM cisplatin or Pt(HexIm)(phen). Digital images of the spheroids were acquired at 0, 3, 6, and 18 h. The orange outline indicates the boundary of the migrated cell area surrounding each spheroid, which was used for quantitative image analysis. (**B**) Tumor spheroids treated or untreated with 1.5 μM cisplatin and Pt(HexIm)(phen) at 3, 6, and 18 h. Migrating areas were measured and plotted as a percentage of initial spheroid area at time 0 (** *p* < 0.01, **** *p* < 0.0001). (**C**) Tumor spheroids treated or untreated with 0.5–1.5 μM cisplatin or Pt(HexIm)(phen) at 18 h. Migrating areas were measured and plotted as a percentage of the control (** *p* < 0.01). All data were expressed as mean values ± standard deviation (SD) of three independent experiments.

Consistently, in 3D spheroids, Pt(HexIm)(phen) suppressed cell migration in a time- and dose-dependent manner, with significant effects observed from 0.5 μM. After 18 h, the migrated area was reduced by ~20% (*p* < 0.0001), whereas cisplatin showed no significant activity (Figure 8).

## 3. Discussion

Pancreatic cancer is a leading cause of cancer-related mortality worldwide and is associated with a very poor prognosis, as reflected by the close correlation between incidence and mortality rates [32,33]. Early diagnosis of pancreatic ductal adenocarcinoma (PDAC) remains challenging due to the lack of early symptoms and the scarcity of highly penetrant risk factors [34]. The high mortality of PDAC is largely attributable to its rapid progression, strong invasiveness, and resistance to current therapies [2]. Cisplatin has demonstrated efficacy in the treatment of several malignancies, including PDAC; however, its clinical use is limited by both intrinsic and acquired resistance [18]. Consequently, there is a growing interest in the development of novel platinum-based compounds with improved anticancer activity [24,35,36,37,38,39].

Organometallic complexes containing 1,10-phenanthroline (phen) have shown promising antitumor activity, particularly in cisplatin-resistant cells. This effect is attributed to the ability of phenanthroline to bind DNA and intercalate between base pairs, thereby interfering with DNA synthesis and repair [40,41]. Consistently, it was reported that platinated phen-containing complexes [Pt(η^1^-C_2_H_4_-OR)(DMSO)(phen)]^+^ (R = Me, Et) exhibit significant cytotoxic activity in PDAC cells, likely due to enhanced intracellular accumulation associated with their lipophilic properties [24,42,43]. More recently, a series of monofunctional cationic platinum(II) complexes containing phenanthroline, including [Pt(NH_3_)(η^1^-C_2_H_4_OEt)(phen)]Cl, [Pt(1-hexyl-1*H*-imidazole)(η^1^-C_2_H_4_OEt)(phen)]Cl, and [Pt(1-hexyl-1*H*-benzo[d]imidazole)(η^1^-C_2_H_4_OEt)(phen)]Cl, were synthesized. Among these, the imidazole-containing complex displayed marked antitumor activity and selectivity toward various cancer cells [25].

The response of various PDAC cell lines to cisplatin, including BxPC-3, MIA PaCa-2, and YAPC cells, frequently reveals a heterogeneous landscape regarding sensitivity and resistance. For instance, BxPC-3 and MIA PaCa-2 cells generally exhibit a higher baseline sensitivity to chemotherapy, whereas YAPC cells display marked resistance, a feature often driven by mesenchymal traits and specific genetic alterations, such as *KRAS* mutations [18,28,29,30]. This differential response is consistent with our previous findings demonstrating that BxPC-3 and Mia PaCa-2 are intrinsically more sensitive to cisplatin than YAPC cells [31]. In that context, cisplatin resistance in YAPC cells was strictly associated with higher basal ERK1/2 activation, and its pharmacological inhibition significantly enhanced cisplatin-induced cytotoxicity, confirming the role of the ERK signaling axis in sustaining this refractory phenotype [31].

Based on these premises, we initially performed a comparative cytotoxic screening across all three cell lines to evaluate whether the structural modifications of Pt(HexIm)(phen) could influence its efficacy against different molecular backgrounds. Remarkably, while Pt(HexIm)(phen) exerted cytotoxic effects across the entire panel, its superior efficacy compared to cisplatin was most pronounced in the highly resistant YAPC cell line. This critical finding prompted us to select YAPC cells as the primary model for further functional and mechanistic analyses, aiming to explore the compound’s capacity to bypass established platinum-resistance mechanisms.

Whether the exceptional efficacy of Pt(HexIm)(phen) in YAPC cells directly involves the modulation or bypass of these ERK-dependent survival pathways remains an intriguing open question that warrants further molecular investigation. However, the fact that the novel complex triggers a rapid, massive apoptotic cascade exactly in the cell line less responsive to cisplatin strongly underscores its potential against treatment-refractory PDAC phenotypes.

Long-term cytotoxic activity was evaluated by clonogenicity assay, and both Pt(HexIm)(phen) and cisplatin had overlapping effects. This trend is consistent with cisplatin’s mechanism of action, based on the formation of DNA adducts and the accumulation of genomic damage, the effects of which become fully evident only after several cell cycles. Consequently, in the long-term clonogenicity assay, the effect of cisplatin was comparable to that of the complex of interest, indicating that, although the latter is more toxic in the initial phases, the final impact on cell proliferation is comparable. The enhanced cytotoxicity of the complex appears to be associated with its higher intracellular accumulation compared to cisplatin. Accordingly, further investigations were conducted to elucidate its proapoptotic and antimigratory properties. Flow cytometry analysis demonstrated a significant increase in late apoptosis in YAPC cells following treatment with the complex. Given the central role of mitochondria in apoptosis, mitochondrial membrane potential (ΔΨ*_M_*) was evaluated as an indicator of mitochondrial function. Under physiological conditions, high ΔΨ*_M_* promotes JC-1 aggregation and red fluorescence emission, whereas depolarization leads to green fluorescence due to the monomeric form of the dye [44]. The red-to-green fluorescence ratio therefore reflects mitochondrial integrity and apoptotic onset. Our results showed that Pt(HexIm)(phen) induced a rapid loss of ΔΨ*_M_*, detectable within minutes of treatment, and more pronounced than that observed with cisplatin. This effect likely reflects the preferential accumulation of lipophilic cationic compounds in mitochondria, which are characterized by a high membrane potential in cancer cells [45,46]. These findings, together with the modulation of apoptosis-regulatory proteins, demonstrate that the activation of the mitochondrial-mediated (intrinsic) apoptotic pathway is a key mechanism underlying the cytotoxicity of the complex [24,47,48,49].

Primary tumor cells must undergo a series of successive stages to acquire a fully metastatic phenotype. During this process, neoplastic cells develop the ability to invade adjacent tissues and migrate through the surrounding environment. The antimigratory and anti-dissemination potential of the compound was evaluated using transwell chemotaxis assays and 3D tumor spheroid models. The complex significantly inhibited cell migration at sublethal concentrations, showing greater efficacy than cisplatin and reducing the migration area in spheroid assays.

A limitation of the present study is that the mechanistic analyses were performed in a single PDAC cell line. Nevertheless, YAPC cells were deliberately selected because of their intrinsic cisplatin-resistant phenotype, which represents a major clinical challenge in pancreatic cancer treatment. To strengthen the biological validation of the compound, its cytotoxic activity was first evaluated across three PDAC cell lines (BxPC-3, Mia PaCa-2, and YAPC), demonstrating consistent antitumor activity despite differences in intrinsic cisplatin sensitivity. Future studies should extend these mechanistic analyses to additional pancreatic cancer models and include non-tumoral pancreatic epithelial cells to further evaluate the selectivity of Pt(HexIm)(phen). Moreover, investigations of Pt–DNA adduct formation, DNA damage responses, cell-cycle regulation, and apoptosis-related signaling pathways are warranted to further elucidate the molecular mechanisms underlying its biological activity.

In summary, the present study demonstrates that the cationic monofunctional organometallic platinum(II) complex [Pt(1-hexyl-1*H*-imidazole)(η^1^-C_2_H_4_OEt)(phen)]Cl exhibits potent antitumor activity across pancreatic ductal adenocarcinoma cell lines with different intrinsic sensitivities to cisplatin. In the cisplatin-resistant YAPC model, Pt(HexIm)(phen) displayed enhanced cytotoxic and antimigratory effects compared with cisplatin and induced mitochondrial membrane depolarization accompanied by modulation of Bax/Bcl-2 expression, caspase-9 activation, and PARP cleavage, consistent with activation of the intrinsic apoptotic pathway. Although the precise molecular targets of the compound remain to be identified, its rapid intracellular accumulation and potent biological activity support further preclinical investigation of Pt(HexIm)(phen) as a promising platinum-based candidate for the treatment of cisplatin-resistant pancreatic ductal adenocarcinoma.

## 4. Materials and Methods

### 4.1. Synthesis of [Pt(1-hexyl-1H-imidazole)(η^1^-C_2_H_4_OEt)(phen)]Cl (Pt(HexIm)(phen))

[Pt(1-hexyl-1*H*-imidazole)(η^1^-C_2_H_4_OEt)(phen)]Cl complexes were synthesized according to a previously reported procedure and provided satisfactory analytical data [25].

Commercially available reagents and solvents were used as received without further purification. Cisplatin was purchased from Sigma-Aldrich Chemical Company (Burlington, MA, USA).

For biological experiments, cisplatin and Pt(HexIm)(phen) stock solutions were freshly prepared in sterile phosphate-buffered saline (PBS, pH 7.4). The stock solutions were further diluted in complete culture medium to the required working concentrations immediately before use. All working solutions were sterile filtered (0.22 μm) prior to cell treatment.

**Figure 9:**
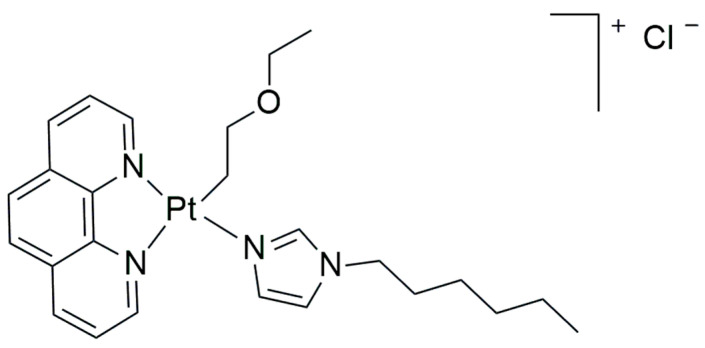
Chemical structure of [Pt(1-hexyl-1*H*-imidazole)(η^1^-C_2_H_4_OEt)(phen)]Cl (Pt(HexIm)(phen)).

The compounds were evaluated over a concentration range of 0.1–100 μM in the SRB cytotoxicity assay, with cisplatin used as a reference compound over the same range. For long-term cytotoxicity, clonogenic survival assays were performed using concentrations 1, 5, 10, and 50 μM. For migration and spheroid assays, sublethal concentrations of 0.5, 1, and 1.5 μM were used for both cisplatin and Pt(HexIm)(phen) (Figure 9).

### 4.2. Cell Culture

Human pancreatic ductal adenocarcinoma YAPC cells (DSMZ, Braunschweig, Germany), a drug-resistant pancreatic cancer cell line, were grown in RPMI 1640 medium (EuroClone, Pero, MI, Italy) supplemented with 10% (*vol*/*vol*) heat-inactivated fetal bovine serum (FBS, EuroClone, Pero, MI, Italy), 2 mM glutamine (EuroClone, Pero, MI, Italy), penicillin (100 U/mL), and streptomycin (100 μg/mL) (EuroClone, Pero, MI, Italy). BxPC-3, and MIA PaCa-2, cells (ATCC, Rockville, MD, USA) were cultured in Dulbecco’s Modified Eagle Medium (DMEM) (4.5 g/L glucose) (EuroClone, Pero, Italy) supplemented with 10% (*vol*/*vol*) heat-inactivated FBS, glutamine 2 mM, penicillin (100 U/mL), and streptomycin (100  μg/mL). Cells were maintained in a humidified incubator containing 5% CO_2_ in air at 37 °C and used for biological analyses upon reaching 70–80% confluence.

### 4.3. Cytotoxic Assay

The viability of pancreatic cancer cells was measured by the sulforhodamine B (SRB) colorimetric assay. A cell suspension (100 µL) was added to each well of a 96-well microtiter plate (10^5^ cell/mL) (Euroclone, Pero, Italy). After an overnight incubation, the cells were treated with different concentrations of Pt(II) compounds, for 18–72 h. Then, 100 μL of ice-cold 10% (*w*/*v*) trichloroacetic acid was added to each well for 30 min at 4 °C. Subsequently, the plates were washed five times with double-distilled water and allowed to air dry overnight. 70 μL of 0.4% (*w*/*v*) SRB solution (SRB; Sigma-Aldrich, Burlington, MA, USA) was added to each well and incubated for 30 min, followed by four washes with 1% (*v*/*v*) acetic acid. Finally, SRB was dissolved in 200 μL of 10 mM unbuffered Tris base solution, and color intensity was measured colorimetrically at 560 nm. Percent cell survival was calculated as the absorbance ratio between treated cells and vehicle-treated control cells. Data are presented as mean ± standard deviation of three independent biological experiments, each performed with eight technical replicates.

### 4.4. Clonogenic Survival Assay

YAPC cells were seeded at a density of 50,000 cell/mL in a 12-well plate (1 mL for well) (Euroclone, Pero, Italy). After overnight incubation, cells were treated with the complexes at increasing concentrations from 1 to 50 µM. After two hours, cells were trypsinized and reseeded at low density (200 cells/mL) in 6-well plates and left to incubate for two weeks. After 15 days of growth, colonies were fixed with a 3:1 mixture of methanol/acetic acid and stained with crystal violet. Only colonies composed of more than 50 cells were evaluated. Three separate experiments were conducted using duplicate samples.

### 4.5. Analysis by ICP-AES (Inductively Coupled Plasma Atomic Emission Spectroscopy)

To determine intracellular platinum accumulation, YAPC cells were incubated with cisplatin or Pt(HexIm)(phen) for 30 min, 1, 3, or 6 h. After treatment, cells were washed twice with ice-cold phosphate-buffered saline (PBS) to remove extracellular platinum and lysed in Radioimmunoprecipitation Assay (RIPA) buffer containing protease and phosphatase inhibitors. Protein concentration was determined using the Bradford colorimetric assay (Bio-Rad, Hercules, CA, USA). Aliquots of each lysate were digested with 1 mL of 67% ultrapure nitric acid at room temperature for 24 h. The digested samples were then diluted to a final volume of 5 mL with ultrapure water, filtered, and analyzed by inductively coupled plasma atomic emission spectroscopy (ICP-AES) using a Thermo iCAP 7000 spectrometer (Thermo Fisher Scientific Inc., Waltham, MA, USA), as previously described [50]. Instrument calibration was performed using platinum standard solutions at 0.001, 0.01, 0.1, and 1 mg/L. Intracellular platinum content is expressed as ng Pt/mg protein.

### 4.6. Annexin V-Fluorescein Isothiocyanate (FITC)/PI Assay

Cell apoptosis was determined using the Annexin V-fluorescein isothiocyanate (FITC) kit (Thermo Fisher Scientific Inc., Waltham, MA, USA). After exposure to Pt(II) compounds at 50 μM of cisplatin and 10 μM of Pt(HexIm)(phen) for 24 h, YAPC cells were harvested, washed twice with PBS, and centrifuged at 1200× *g* for 5 min at room temperature. Subsequently, the cell pellet was resuspended in Annexin V-FITC and propidium iodide (PI) solutions. After incubation for 15 min at room temperature in the dark, additional Annexin V binding buffer (10 mM Hepes, 140 mM NaCl, and 2.5 mM CaCl_2_) was added to each sample. Cells were analyzed using a flow cytometer (Partec CyFlow^®^ space) and FloMax software (Sysmex Partec GmbH, Görlitz, Germany).

### 4.7. Mitochondrial Membrane Potential (JC-1) Assay

YAPC cells were cultured and incubated with 5 μg/mL JC-1 (5,5′,6,6′-tetrachloro-1,1′,3,3′-tetraethylbenzimi- dazolylcarbocyanine iodide) fluorescent probe (Enzo Life Sciences, Farmingdale, NY, USA) before being treated with 50 μM cisplatin or 10 μM Pt(HexIm)(phen). The fluorescence of JC-1 was measured using a Jasco FP-750 Spectrofluorometer (JASCO Corporation, Tokyo, Japan). Fluorescence readings were taken at various time points during each experiment. The ratio of JC-1 fluorescence intensity at 590 and 520 nm was calculated for each time point and used as a qualitative measure of mitochondrial membrane potential (ΔΨ*_M_*).

### 4.8. Fluorescence Microscopy: Double Staining of Nuclei (DAPI) and Mitochondria (JC-1)

The induction of apoptosis in YAPC cells was assessed using fluorescence microscopy, with JC-1 and DAPI (4′,6-diamidino-2-phenylindole) staining. The cells were treated with 50 μM of cisplatin and 10 μM of Pt(HexIm)(phen) for 24 h and then stained with 5 μg/mL JC-1 following the protocol described in the previous paragraph. After fixation with 4% (*w*/*v*) paraformaldehyde, the cells were stained with 1 μg/mL DAPI. The EVOS XL Cell Imaging System microscope (Thermo Fisher Scientific, Waltham, MA, USA) was used to analyze cell fluorescence.

### 4.9. Preparation of Subcellular Fractions and Western Blotting

Subcellular fractions were obtained as previously reported [51]. Western blotting analysis, immunodetection, and densitometric analysis were performed as previously described [52].

### 4.10. Transwell Chemotaxis and Migration Assay

1 × 10^5^ cells per insert, in 1% serum-supplemented medium, were seeded in the upper chamber of a Transwell insert (8 μm pore diameter; Corning Inc., Corning, NY, USA), while 10% FBS (fetal bovine serum) medium was added to the lower chamber as a chemoattractant. After adhering the cells to the bottom of the insert for 1 h, they were treated with increasing sublethal concentrations (0.5–1.5 μM) of Pt(II) complexes. After incubation at 37 °C, 5% CO_2_ for 24 h, the Transwell chambers were removed, and the culture medium in the well was discarded and washed with calcium-free PBS. The cells were then fixed with 4% PFA for 15 min and stained with 0.1% crystal violet for 30 min. Non-migrating cells were removed with a cotton swab. Images were collected under a microscope (Evos XL, Core; Invitrogen) and subsequently quantified using ImageJ software version 1.54t.

### 4.11. 3D Spheroid-Based Migration Assay

YAPC tumor spheroids (TS) were generated according to the liquid-overlay technique, as previously reported [42]. Briefly, a cell suspension (2.5 × 10^5^ cells/mL) was cultured under non-adherent conditions to promote spontaneous spheroid formation. After 4 days of incubation, compact spheroids were obtained and individually transferred to gelatin-coated 96-well plates) (Euroclone, Pero, Italy) (one spheroid per well in a final volume of 200 μL). Images were acquired at 0, 3, 6, 18, and 24 h using an inverted microscope. The area covered by cells migrating from the spheroid core was quantified using Photoshop CS6 software version 13.0 (Adobe, San Jose, CA, USA). Migration was expressed as a percentage of the initial spheroid area according to the formula: (migrated area at time x/migrated area at time 0) × 100. All experiments were performed in three independent biological replicates.

### 4.12. Statistical Analysis

Statistical analyses were conducted using GraphPad Prism 8 software (GraphPad Software, San Diego, CA, USA). Results are shown as means ± SD. Statistical analysis was carried out using ANOVA and as indicated, post hoc tests (Bonferroni-Dunn) were also performed. A *p* value < 0.05 was considered to achieve statistical significance.

## 5. Conclusions

The present study demonstrates that Pt(HexIm)(phen) exerts significant anticancer activity against human drug-resistant YAPC pancreatic ductal adenocarcinoma cells, a clinically relevant model of treatment-refractory disease. The compound induced enhanced intracellular accumulation and mitochondrial depolarization, accompanied by significant cytotoxic and antimigratory effects. The observed mitochondrial depolarization, together with the modulation of apoptosis-regulatory proteins, indicates that the activation of the mitochondrial-mediated apoptotic pathway is a key mechanism underlying the biological activity of Pt(HexIm)(phen). In addition, the ability of the compound to impair both migratory behavior and spheroid dissemination highlights its potential to interfere with processes associated with tumor progression.

Overall, the results indicate that the incorporation of a lipophilic imidazole ligand into the platinum (II) scaffold may enhance cellular uptake and biological activity in aggressive and chemoresistant pancreatic cancer cells. Further studies are warranted to clarify its molecular targets, evaluate its selectivity toward non-tumoral pancreatic cells, and confirm its efficacy in additional experimental models. Taken together, the present findings identify Pt(HexIm)(phen) as a promising platinum-based compound for the targeting of chemoresistant pancreatic ductal adenocarcinoma cells.

## Data Availability

All data supporting the findings of this study are included within the article.

## Abbreviations

The following abbreviations are used in this manuscript:

DAPI	4′,6-diamidino-2-phenylindole
DMSO	Dimethyl sulfoxide
DNA	DeoxyriboNucleic Acid
FBS	Fetal bovine serum
IC_50_	Half-maximal inhibitory concentrations
ICP-AES	Inductively coupled plasma atomic emission spectroscopy
JC-1	5,5′,6,6′-tetrachloro-1,1′,3,3′-tetraethylbenzimi- dazolylcarbocyanine iodide
PDAC	Pancreatic ductal adenocarcinoma
Pt(HexIm)(phen)	[Pt(1-hexyl-1*H*-imidazole)(η^1^-C_2_H_4_OEt)(phen)]Cl
SRB	Sulforodhamine B
YAPC	Pancreatic ductal adenocarcinoma cells
ΔΨ_*M*_	Mitochondrial membrane potential
